# Supplementary material for: Modulatory effects of dynamic fMRI-based neurofeedback on emotion regulation networks in adolescent females
Source: Neuroimage. 2020 Oct 15;220:117053. doi: 10.1016/j.neuroimage.2020.117053 (PMC7573536; doi:10.1016/j.neuroimage.2020.117053)
Supplement: Multimedia component 1 [file mmc1.pdf]

Supplementary Materials for:
Modulatory effects of dynamic fMRI-based neurofeedback on emotion regulation networks in adolescent females

Catharina Zich, Nicola Johnstone, Michael Lührs, Stephen Lisk, Simone P W Haller, Annalisa Lipp, Jennifer Y F Lau, Kathrin Cohen Kadosh

Supplementary Methods

MRI data acquisition

Before the experimental tasks a high-resolution structural scan was obtained from each subject using a T1-weighted magnetization-prepared rapid-acquisition gradient echo (MPRAGE) sequence (TR = 1900 ms, TE = 3.97 ms, filp angle = 8°, slice thickness 1 mm, in-plane resolution 1 x 1 mm, orientation = sagittal).

Functional data were recorded using 2D multiband gradient echo planer imaging (Todd et al., 2016) (TR = 933 ms, TE = 33.40 ms, flip angle = 64°, slice thickness 2 mm, in-plane resolution 2 x 2 mm, orientation = transversal, 72 slices, multi-band factor = 6).

For **Experiment 1** and **2** the localizer comprised 570 volumes and each of the four NF runs comprised 310 volumes. For **Experiment 3** the localizer comprised 300 volumes and each of the three NF runs comprised 330 volumes. At the beginning of the localizer and each NF run ten additional volumes were acquired, but not analysed, to avoid saturation effects.

For **Experiment 3** we collected MRS data. MRS data were acquired using a semi-LASER sequence (Scheenen et al., 2008) (32 averages, TR = 3500 ms, TE = 28 ms, voxel size = 20 x 20 x 20 mm) using VAPOR (variable power RF pulses with optimized relaxation delays) water suppression (Tkác et al., 1999). Two voxels of interest (VOI) were manually positioned. One VOI was positioned at the same position as the PFC ROI, i.e. local maximum of the GLM t-statistics of the brain activity during the localizer (the sum of the three contrasts: appraisal > fixation, reappraisal > fixation, reappraisal > appraisal) within the left dorsolateral and medial PFC. The other VOI, the anterior cingulate cortex (ACC) VOI, was centred on the interhemispheric fissure on the coronal plane superior to the frontal aspect of the corpus callosum on the sagittal plane.

Neurofeedback task – Instructions

All participants were blind to their NF implementation and were instructed as follows: *"You will see a thermometer with a green rim on the screen. The red bars show how much the regions that are important for emotions are active. Your job is to get these regions as active as possible! So try to get this thermometer up as much as possible. Similar to the task before, try to control your thoughts towards a positive feeling. When the thermometer does not have a green rim, the thermometer is not working. However, even if the thermometer is not working, your task will be the same and we are still measuring how much your brain is active. The two different thermometers will alternate.*

When the thermometer has a green rim, it might need some time to properly estimate the brain activity. This leads to a delay in the thermometer display." Debriefing revealed that, in order to change their thoughts and emotions towards a happier mood, participants thought about certain people, things that happened in the past and things that they would like to happen in the future.

Supplementary Results

NF implementations differentially modulate the frequency of negative PFC-amygdala partial correlations

The observed differences in fc between fc-NF and no-NF as illustrated in Supplementary Fig. 1 are most likely due to changes in the frequency of negative partial correlations (secondary measure, **Supplementary Fig. 2a**).

The relationship of fc-NF and fc was assessed by correlating the frequency of negative partial correlations at each of the 20 volumes with the number of fc-NF volumes within the correlation window (**Supplementary Fig. 2b**). For the negative NF implementation and the positive NF implementation, the frequency of negative partial correlations and the number of fc-NF volumes in the correlation window were related negatively at trend level (negative NF implementation: $r_{(38)} = -.31$, $p = .05$; positive NF implementation: $r_{(38)} = -.32$, $p = .05$). Contrary, for the weighted negative NF implementation a positive relationship was observed ($r_{(38)} = .47$, $p < .01$), indicating that a higher number of fc-NF volumes within the correlation window equalled a higher frequency of negative partial correlation.

The relationship between the frequency of negative partial correlations and the number of fc-NF volumes in the correlation window was significantly different for the weighted negative NF implementation in comparison to the other two NF implementations (both *p*'s $< .001$), whereby the other two NF implementations did not differ regarding this aspect ($p = .96$).

NF implementations differentially modulate the frequency of correlation scenarios

One limitation of using a fc-NF approach that is based on correlational measures is that we cannot directly infer the direction of change within each region. In other words, a negative correlation exits when variable 1 increases and variable 2 decreases, or when variable 1 decreases and variable 2 increases. However, given the top-down PFC regulation of amygdala reactivity the main goal of PFC-amygdala fc-NF constitutes PFC increase and amygdala decrease.

To understand the nature of the results depicted in Supplementary Fig. 1 and 2, we assigned each negative correlation to one out of four scenarios (see **Supplementary Fig. 5** for details). **Supplementary Fig. 3a** illustrates the percentage of each scenario for fc-NF and no-NF mini-blocks.

As above, we further investigated the relationship between the number of fc-NF volumes within the correlation window and the frequency of scenario 1, i.e. PFC up-regulation & amygdala down-regulation, and of scenario 2, i.e. PFC down-regulation & amygdala up-regulation, at each volume (**Supplementary Fig. 3b**). For the negative NF implementation and the weighted negative NF implementation the number of fc-NF volumes in the correlation window was positively related to the frequency of scenario 1 (negative NF implementation: $r_{(38)} = 0.39$, $p = .01$; weighted negative NF implementation: $r_{(38)} = .70$, $p < .001$) and negatively related to the frequency of scenario 2 (negative NF implementation: $r_{(38)} = -.50$, $p < .01$; weighted negative NF implementation: $r_{(38)} = -.64$, $p < .001$). In contrast, for the positive NF implementation the pattern was reversed, i.e. the number of fc-NF volumes in the correlation window was negatively related to the frequency of scenario 1 ($r_{(38)} = -.44$, $p < .01$) and positively related to the frequency of scenario 2 ($r_{(38)} = .55$, $p < .001$). This suggests that only NF implementations reinforcing negative PFC-amygdala fc support the task-relevant negative fc pattern.

Further, we compared the two negative NF implementations with regard to the strength of the relationship between the number of fc-NF volumes in the correlation window and the frequency of scenario 1. We found a significant difference, indicating that the relationship is strongest for the weighted negative NF implementation.

Supplementary Discussion

Length of the correlation window

We chose a window length of 20 volumes based on a previous study by Zilverstand and colleagues (Zilverstand et al., 2014), who found that the fc measures in a well-controlled, simple motor task were weaker and less reliable for a shorter (12 s) window but not qualitatively different form a longer (26 s) window. Given the higher reliability of the longer correlation windows, we matched the size of our time-windows with the length of the mini-blocks. This allowed us to directly assess the effect of fc-NF as a function of the ratio of fc-NF : no-NF volumes in the correlation window. We found that the PFC-amygdala fc (Supplementary Fig. 1, Fig. 2a,c), frequency of negative partial correlations (Supplementary Fig. 2, Fig. 2b,d), and frequency of scenarios describing different patterns of activation changes (Supplementary Fig. 3) changed as a function of fc-NF volumes within a correlation window. This finding is not only of theoretical interest, but also has important practical implications. Specifically, it suggests that with regards to clinical applications the relation between task window and correlation window should be adapted, so that the length of the task window exceeds the correlation window, in order to further enhance the fc-NF-effect and practice-related changes.

Supplementary Figures

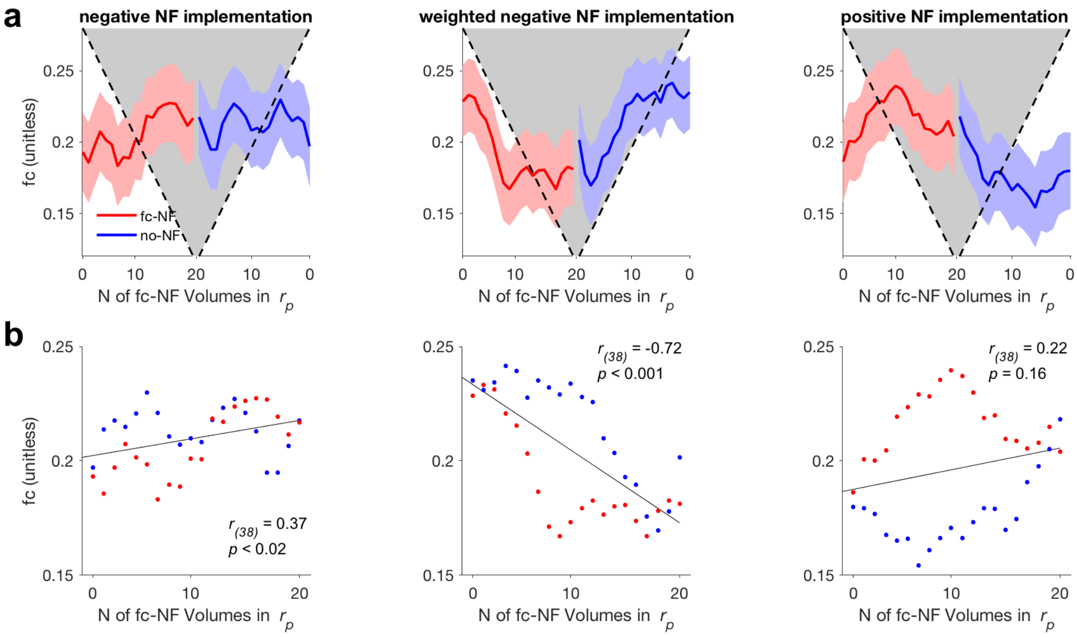

Supplementary Fig. 1 Difference in average functional connectivity (fc) between fc-NF (red) and the no-NF (blue) for each NF implementation (N = 5 per NF implementation). **(a)** Means and standard error for the average fc, i.e. partial correlations r_p, at each volume within a mini-block. Fc is averaged across mini-blocks within one condition (fc-NF, no-NF), NF runs (1, 2, 3, 4) and individuals within one NF implementation (negative NF implementation, weighted negative NF implementation, positive NF implementation). Number of fc-NF volumes in the correlation window (N of fc-NF Volumes in r_p) is illustrated in form of a grey triangle. **(b)** Relationship between average fc at each volume and the number of fc-NF volumes within the correlation window.

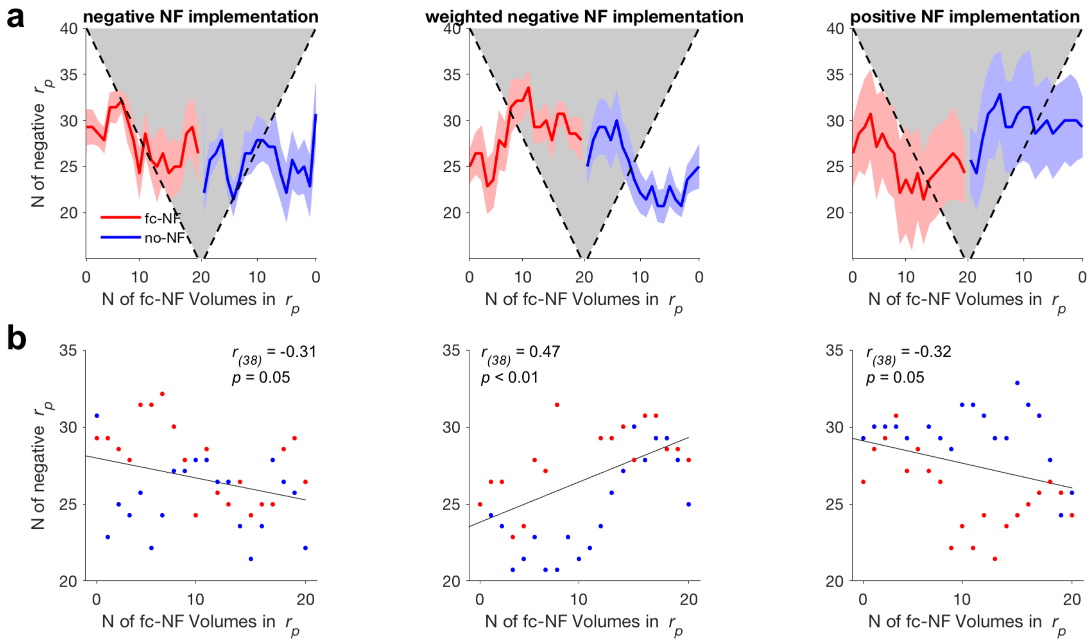

Supplementary Fig. 2 As Supplementary Fig. 1, but for the frequency of negative partial correlations (N of negative r_p).

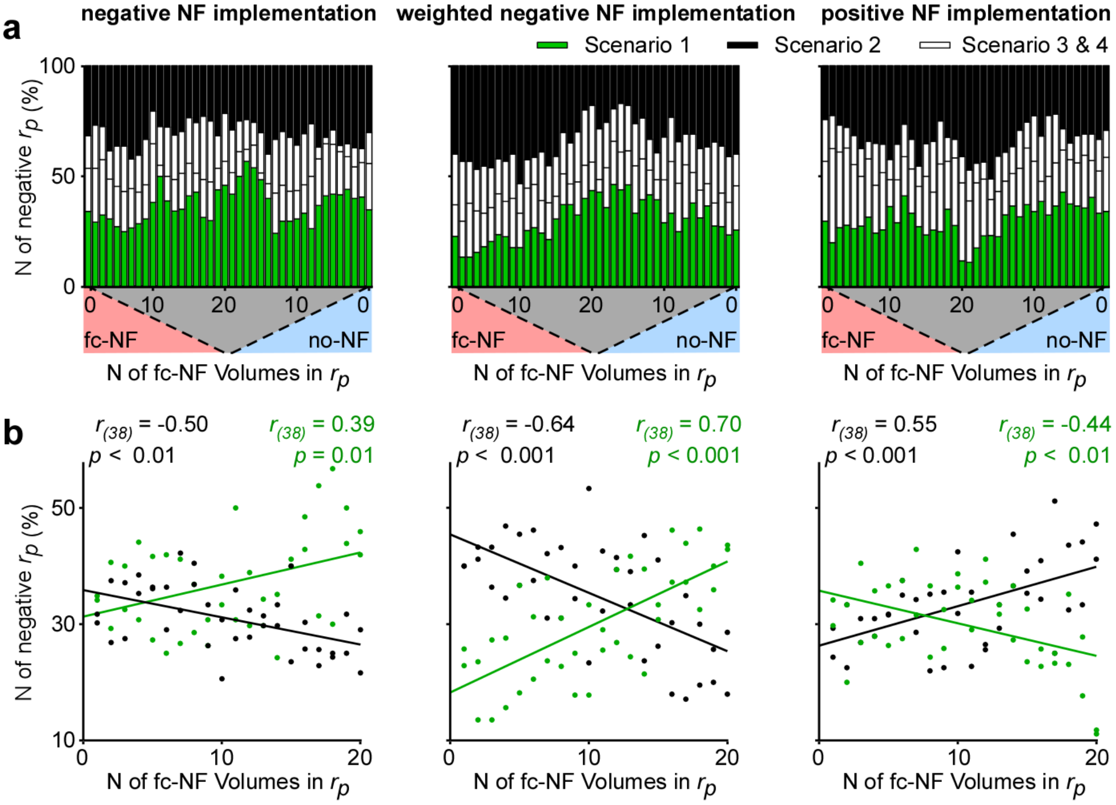

Supplementary Fig. 3 Direction of activity change within the negative partial correlations for each NF implementation (*N* = 5 per implementation). **(a)** Percentage of negative partial correlations between PFC and amygdala for scenario 1 (PFC up-regulation & amygdala down-regulation; green), scenario 2 (PFC down-regulation & amygdala up-regulation; black) or scenario 3 and 4 (white). Percentages are averaged across mini-blocks within one condition (fc-NF, no-NF), NF runs (1, 2, 3, 4) and individuals within one NF implementation (negative NF implementation, weighted negative NF implementation, positive NF implementation). The number of fc-NF volumes in the correlation window is illustrated in form of a grey triangle. **(b)** Relationships between the number of fc-NF volumes within the correlation window (N of fc-NF Volumes in r_p) and frequency of scenario 1 (PFC up-regulation & amygdala down-regulation; green) or scenario 2 (PFC down-regulation & amygdala up-regulation; black).

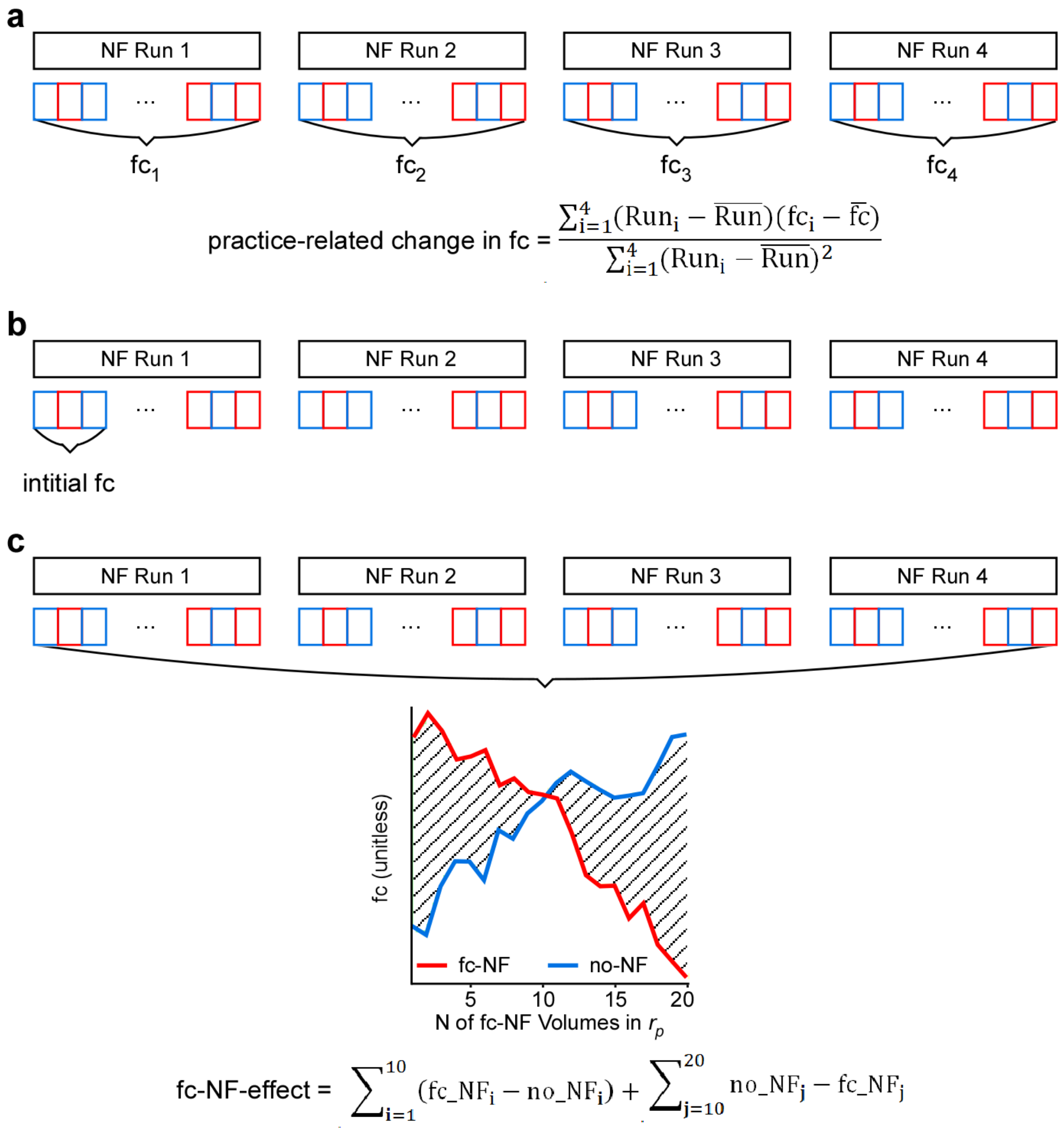

Supplementary Fig. 4 Illustration of quantification of neural measures. (**a**) Visualization of practice-related change in fc, i.e. slope of the linear regression for the average fc within each NF run. (**b**) Illustration of initial fc, i.e. average fc of the first two mini-blocks. (**c**) Visualization of the fc-NF-effect (hatched) on exemplary data from one subject. Due to the dynamic character of the fc-NF, the fc-NF-effect is composed of two parts. For fc-NF mini-bocks (red) the ratio of fc-NF : no-NF volumes in the correlation window is 1:19 at the first volume of the mini-block, 2:18 at the second volume of the mini-block and 20:0 at the last volume of the mini-block. Consequently, in fc-NF mini-blocks volumes 1 to 10 are dominated by no-NF volumes, and volumes 11 to 20 are dominated by fc-NF volumes. Exactly the opposite is the case for no-NF mini-blocks.

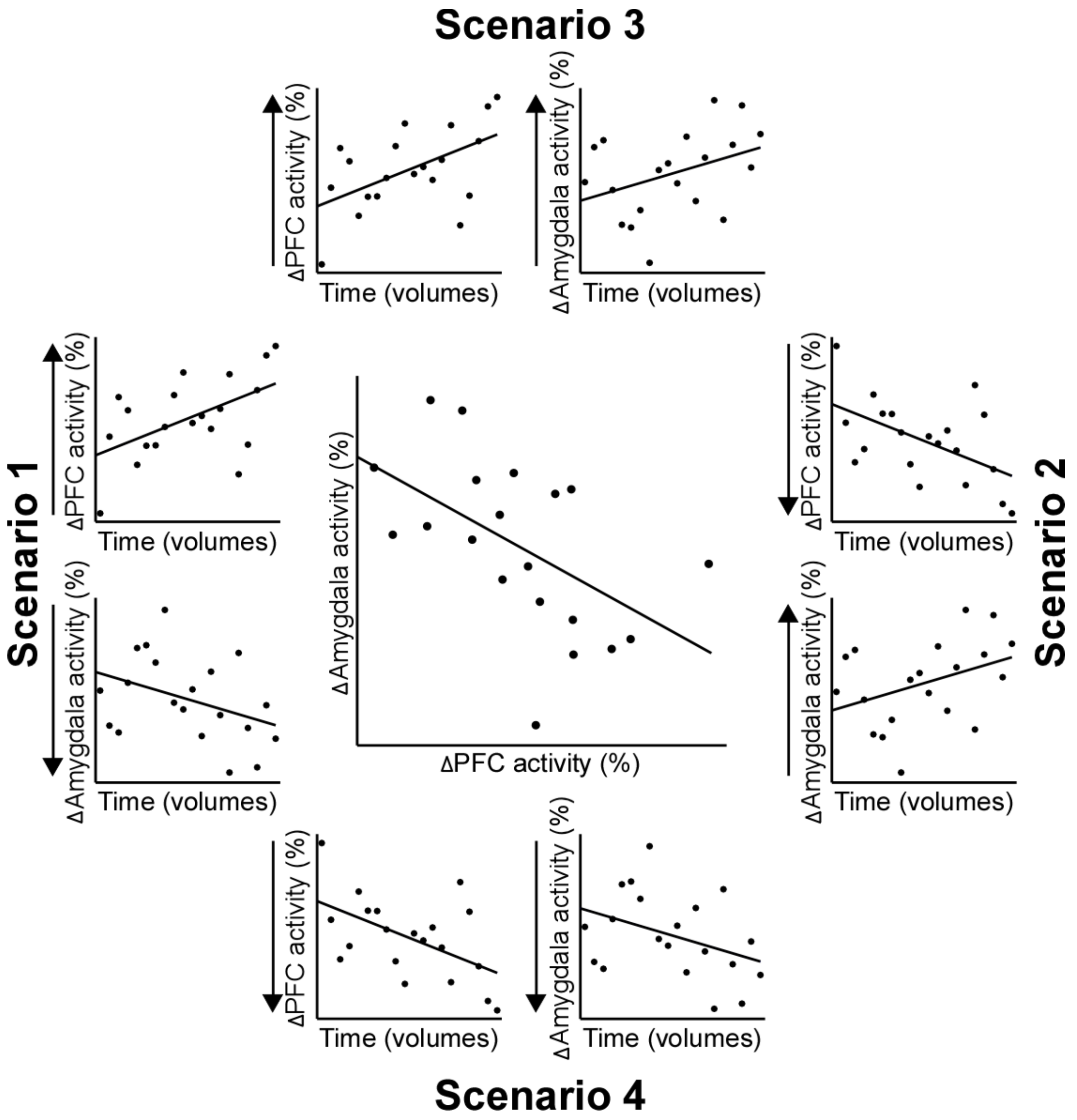

Supplementary Fig. 5 Schematic representation of the procedure used to quantify the direction of activity change within a negative partial correlation. **Centre**: Exemplary negative partial correlation between PFC and amygdala activity accounting for the 'activity' in the CST. The direction of activity change within this correlation was determined for the PFC and the amygdala separately. In detail, both, the PFC and the amygdala, were correlated separately with time while accounting for CST 'activity'. Theoretically four different scenarios are possible: scenario 1 – PFC up-regulation & amygdala down-regulation, scenario 2 – PFC down-regulation & amygdala up-regulation, scenario 3 – PFC & amygdala up-regulation and scenario 4 – PFC & amygdala down-regulation.

Supplementary Tables

Supplementary Table 1 Emotional/metacognitive measures for **Experiment 1**.

Pre	*Negative NF implementation (N=5)*	*Weighted negative NF implementation (N=5)*	*Positive NF implementation (N=5)*
$CERQ_{pre}$	*M = 59.50; SD = 9.26*	*M = 57.40; SD = 7.33*	*M = 53.65; SD = 10.65*
MFQ_{pre}	*M = 2.60; SD = 2.07*	*M = 1.50; SD = 1.91*	*M = 2.20; SD = 1.71*
SAS-S	*M= 46.00; SD = 13.41*	*M = 48.00; SD = 11.22*	*M = 56.80; SD = 14.62*
$STAI\text{-}S_{pre}$	*M = 31.40; SD = 8.91*	*M = 23.00; SD = 6.38*	*M = 36.80; SD = 5.22*
STAI-T	*M = 35.20; SD = 7.92*	*M = 35.80; SD = 8.56*	*M = 38.00; SD = 9.92*
TCQ_{pre}	*M = 65.75; SD = 4.27*	*M = 67.20; SD = 9.26*	*M = 59.60; SD = 7.50*
$TCAQ_{pre}$	*M = 79.40; SD = 14.62*	*M = 82.00; SD = 10.42*	*M = 75.60; SD = 13.63*

CERQ = Emotion Regulation Questionnaire; *MFQ* = Mood and Feelings Questionnaire; *SAS-S* = Social Anxiety Scale for Adolescents; *STAI-S, STAI-T* = State-Trait Anxiety Inventory; *TCQ* = Thought Control Questionnaire; *TCAQ* = Cognitive Thought Control Ability Questionnaire

Supplementary Table 2 Emotional/metacognitive measures for **Experiment 2**.

	Pre	*Post*	*Difference*
CERQ	*N = 27; M = 60.52; SD = 11.09*	*N = 27; M = 60.93; SD = 11.18*	$t_{(26)}$ = -.442; p = .662
MFQ	*N = 25; M = 5.44; SD = 4.81*	*N = 27; M = 5.56; SD = 4.56*	$t_{(24)}$ = -.778; p = .444
SAS-S	*N = 27; M= 49.15; SD = 15.01*		
STAI-S	*N = 24; M = 32.79; SD = 8.61*	*N = 27; M = 31.78; SD = 9.83*	$t_{(23)}$ = 1.784; p = .088
STAI-T	*N = 27; M = 43.52; SD = 11.49*		
TCQ	*N = 27; M = 61.04; SD = 8.67*	*N = 27; M = 60.70; SD = 8.99*	$t_{(26)}$ = .251; p = .804
TCAQ	*N = 23; M = 78.91; SD = 15.37*	*N = 27; M = 79.85; SD = 14.77*	$t_{(22)}$ = .041; p = .968

CERQ = Emotion Regulation Questionnaire; *MFQ* = Mood and Feelings Questionnaire; *SAS-S* = Social Anxiety Scale for Adolescents; *STAI-S, STAI-T* = State-Trait Anxiety Inventory; *TCQ* = Thought Control Questionnaire; *TCAQ* = Cognitive Thought Control Ability Questionnaire

Supplementary Table 3: Emotional/metacognitive measures for a subsample of **Experiment 2** (negative level, i.e. change in the desired direction, *N*=13).

	Pre	*Post*	*Difference*
CERQ	*N = 13; M = 62.92; SD = 12.43*	*N = 13; M = 64.15; SD = 12.43*	$t_{(12)} = -.854; p = .410$
MFQ	*N = 12; M = 4.92; SD = 5.00*	*N = 13; M = 4.92; SD = 4.92*	$t_{(11)} = -.561; p = .586$
SAS-S	*N = 13; M= 52.15; SD = 13.45*		
STAI-S	*N = 13; M = 31.83; SD = 9.04*	*N = 13; M = 30.85; SD = 7.86*	$t_{(12)} = .616; p = .551$
STAI-T	*N = 13; M = 42.54; SD = 12.14*		
TCQ	*N = 13; M = 62.69; SD = 8.60*	*N = 13; M = 60.77; SD = 10.69*	$t_{(12)} = 1.402; p = .133$
TCAQ	*N = 11; M = 81.27; SD = 15.38*	*N = 13; M = 80.54; SD = 15.01*	$t_{(10)} = 1.258; p = .237$

CERQ = Emotion Regulation Questionnaire; *MFQ* = Mood and Feelings Questionnaire; *SAS-S* = Social Anxiety Scale for Adolescents; *STAI-S, STAI-T* = State-Trait Anxiety Inventory; *TCQ* = Thought Control Questionnaire; *TCAQ* = Cognitive Thought Control Ability Questionnaire

Supplementary Table 4: Emotional/metacognitive measures for a subsample of **Experiment 2** (positive level, i.e. change in the undesired direction *N*=14).

	Pre	*Post*	*Difference*
CERQ	*N = 14; M = 58.29; SD = 9.60*	*N = 14; M = 57.93; SD = 9.34*	$t_{(13)} = .302; p = .768$
MFQ	*N = 13; M = 5.92; SD = 4.79*	*N = 14; M = 6.14; SD = 4.29*	$t_{(12)} = -.519; p = .613$
SAS-S	*N = 14; M= 46.36; SD = 16.33*		
STAI-S	*N = 12; M = 33.75; SD = 8.43*	*N = 14; M = 32.64; SD = 11.59*	$t_{(11)} = 1.170; p = .115$
STAI-T	*N = 14; M = 44.43; SD = 11.24*		
TCQ	*N = 14; M = 59.50; SD = 8.76*	*N = 14; M = 61.57; SD = 7.38*	$t_{(13)} = -.965; p = .352$
TCAQ	*N = 12; M = 76.75; SD = 15.70*	*N = 14; M = 79.21; SD = 15.08*	$t_{(11)} = -1.105; p = .293$

CERQ = Emotion Regulation Questionnaire; *MFQ* = Mood and Feelings Questionnaire; *SAS-S* = Social Anxiety Scale for Adolescents; *STAI-S, STAI-T* = State-Trait Anxiety Inventory; *TCQ* = Thought Control Questionnaire; *TCAQ* = Cognitive Thought Control Ability Questionnaire

Supplementary Table 5 Emotional/metacognitive measures for **Experiment 3**.

	Pre	*Post*	*Difference*
CERQ	*N = 19; M = 58.16; SD = 9.00*	*N = 19; M = 60.84; SD = 11.08*	$t_{(18)}$ = -1.326; p = .202
MFQ	*N = 19; M = 6.21; SD = 5.72*	*N = 19; M = 6.42; SD = 5.82*	$t_{(18)}$ = -.200; p = .844
SAS-S	*N = 19; M= 48.00; SD = 15.37*		
STAI-S	*N = 19; M = 31.42; SD = 6.01*	*N = 19; M = 29.00; SD = 5.85*	$t_{(18)}$ = 1.490; p = .153
STAI-T	*N = 19; M = 42.42; SD = 10.70*		
TCQ	*N = 19; M = 60.42; SD = 7.52*	*N = 19; M = 62.74; SD = 7.90*	$t_{(18)}$ = -1.180; p = .254
TCAQ	*N = 19; M = 74.47; SD = 18.20*	*N = 19; M = 75.58; SD = 19.27*	$t_{(18)}$ = -.045; p = .657

CERQ = Emotion Regulation Questionnaire; *MFQ* = Mood and Feelings Questionnaire; *SAS-S* = Social Anxiety Scale for Adolescents; *STAI-S, STAI-T* = State-Trait Anxiety Inventory; *TCQ* = Thought Control Questionnaire; *TCAQ* = Cognitive Thought Control Ability Questionnaire

References

Scheenen, T.W.J., Klomp, D.W.J., Wijnen, J.P., Heerschap, A., 2008. Short echo time1H-MRSI of the human brain at 3T with minimal chemical shift displacement errors using adiabatic refocusing pulses. Magn. Reson. Med. 59, 1–6. https://doi.org/10.1002/mrm.21302

Tkác, I., Starcuk, Z., Choi, I.Y., Gruetter, R., 1999. In vivo 1H NMR spectroscopy of rat brain at 1 ms echo time. Magn. Reson. Med. 41, 649–56.

Todd, N., Moeller, S., Auerbach, E.J., Yacoub, E., Flandin, G., Weiskopf, N., 2016. Evaluation of 2D multiband EPI imaging for high-resolution, whole-brain, task-based fMRI studies at 3T: Sensitivity and slice leakage artifacts. Neuroimage 124, 32–42. https://doi.org/10.1016/j.neuroimage.2015.08.056

Zilverstand, A., Sorger, B., Zimmermann, J., Kaas, A., Goebel, R., 2014. Windowed Correlation: A Suitable Tool for Providing Dynamic fMRI-Based Functional Connectivity Neurofeedback on Task Difficulty. PLoS One 9, e85929. https://doi.org/10.1371/journal.pone.0085929
